# Invasions and Extinctions Reshape Coastal Marine Food Webs

**DOI:** 10.1371/journal.pone.0000295

**Published:** 2007-03-14

**Authors:** Jarrett E. Byrnes, Pamela L. Reynolds, John J. Stachowicz

**Affiliations:** 1 Center for Population Biology, University of California, Davis, California, United States of America; 2 Bodega Marine Laboratory, Bodega Bay, California, United States of America; Dalhousie University, Canada

## Abstract

The biodiversity of ecosystems worldwide is changing because of species loss due to human-caused extinctions and species gain through intentional and accidental introductions. Here we show that the combined effect of these two processes is altering the trophic structure of food webs in coastal marine systems. This is because most extinctions (∼70%) occur at high trophic levels (top predators and other carnivores), while most invasions are by species from lower trophic levels (70% macroplanktivores, deposit feeders, and detritivores). These opposing changes thus alter the shape of marine food webs from a trophic pyramid capped by a diverse array of predators and consumers to a shorter, squatter configuration dominated by filter feeders and scavengers. The consequences of the simultaneous loss of diversity at top trophic levels and gain at lower trophic levels is largely unknown. However, current research suggests that a better understanding of how such simultaneous changes in diversity can impact ecosystem function will be required to manage coastal ecosystems and forecast future changes.

## Introduction

The biodiversity of ecosystems around the world is being altered by species loss due to extinction from human activities [1] and gain through intentional and accidental introductions [2]. Although a number of studies have recently considered the consequences of diversity loss for ecosystem functioning [see reviews by 3], [4], few of these consider the effects of realistic diversity change scenarios [but see 5], [6]. At the regional scale, the gain of species most often equals or outpaces the number lost due to extinction [2], suggesting that extinctions and invasions might offset one another with little net change in diversity.

Because different processes drive extinctions and invasion (e.g., overfishing versus ballast water transport), the *types* of species being gained and lost might differ, however. For example, extinctions due to anthropogenic stressors such as overfishing and climate change are thought to impact higher trophic levels first [7], [8]. Such a re-organization of trophic structure (i.e., trophic skew *sensu* Duffy [9]) may result in major changes to ecosystem structure and function, even if the total number of species in a region remains constant or even increases. These changes can impact a wide variety of ecosystem level processes [10], [11] such as the total biomass and production and their distribution among trophic levels.

**Figure 1:**
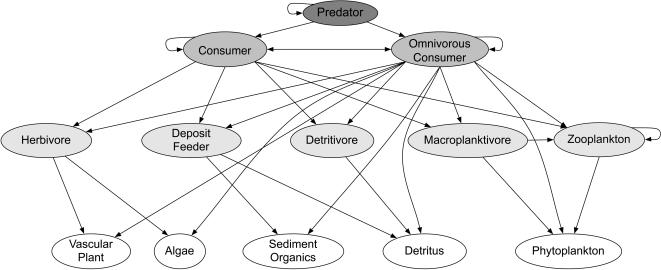
Food web showing the connections between all trophic functional groups. Arrows represent one group consuming the group to which the arrow points. Shading indicates trophic level (none = 1, light = 2, moderate = 3, dark = 4).

Here we consider how modern invasions and extinctions have together changed the architecture of marine food webs by comparing the trophic distribution of invasions and extinctions. In a recent analysis for a single region, the Wadden Sea, the taxonomic distribution of extinctions and invasions differed even though overall richness was relatively unchanged [12]. However, the generality of this trend is not clear, and this study only categorized species by coarse taxonomic group, which often does not correlate with ecological function. In this paper, we classified all species in lists of global and regional marine species extinctions [13] and invasions from lists for San Francisco Bay [14], Australia [15], The Gulf of the Farallones, and the Wadden Sea [16], [17] by trophic level and feeding mode (Fig. 1, see Methods for details). We then compared the distribution of species among trophic groups in each exotic species list with that of global and regional extinctions to assess whether the trophic distribution of species additions matched that of species deletions and to estimate the net change in species richness of each trophic level and functional feeding group. Our results suggest that invasions are biased towards lower trophic levels whereas extinctions occur higher in the food web. We discuss the potential implications of these changes in trophic skew [9] for marine ecosystems.

## Results

**Figure 2:**
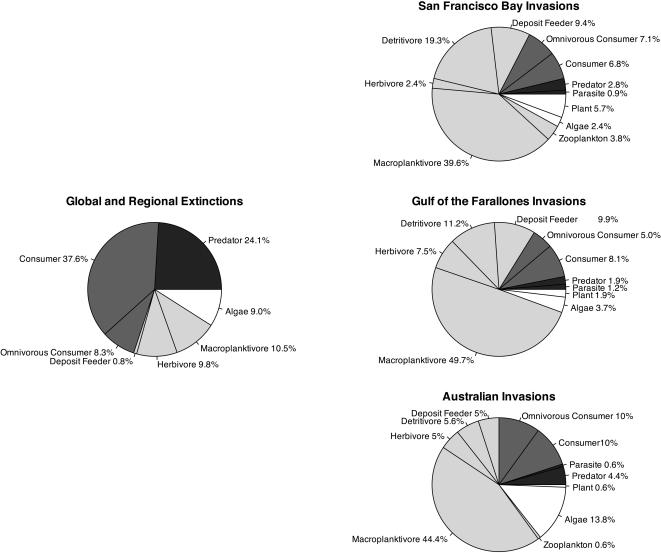
Trophic skew in regional invasions versus combined global and regional extinctions broken down by percentage of species in each trophic group. Colors indicate trophic level (white = 1, light grey = 2, dark grey = 3, black = 4). Extinctions are skewed towards trophic levels 3 and 4 (secondary consumers and predators) while invasions are skewed towards trophic level 2 (primary consumers).

The top two trophic levels (secondary consumers and predators) contained 70% of the 133 documented global and regional marine extinctions (Fig. 2). Conversely, in each invasion list, approximately 70% of exotic species belonged to trophic level two, the majority of which were suspension feeders that directly consume plankton, deposit feeders that consume sediment organic material, or detritivores that consume decaying organic matter (Fig. 2). A comparison of the trophic distribution of extinctions with each invasion data set shows that species loss is skewed toward species from higher trophic groups relative to species gain, which is skewed towards lower order consumers (San Francisco Bay χ^2^_10_ = 163.03 p<0.0001, Gulf of the Farallones National Marine Sanctuary χ^2^_9_ = 126.64 p<0.0001, Australia χ^2^_10_ = 90.02 p<0.0001). The functional groups most responsible for this skew are top predators (24.1% of extinctions but 6.1% of invasions on average), secondary consumers (37.6% of extinctions but 2.2% of invasions), and suspension feeding macroplanktivores (10.5% of extinctions but 44.6% of invasions). Changes in primary producers from invasions roughly match those due to extinctions. The distribution of introduced species among functional groups is remarkably similar among regions.

**Figure 3:**
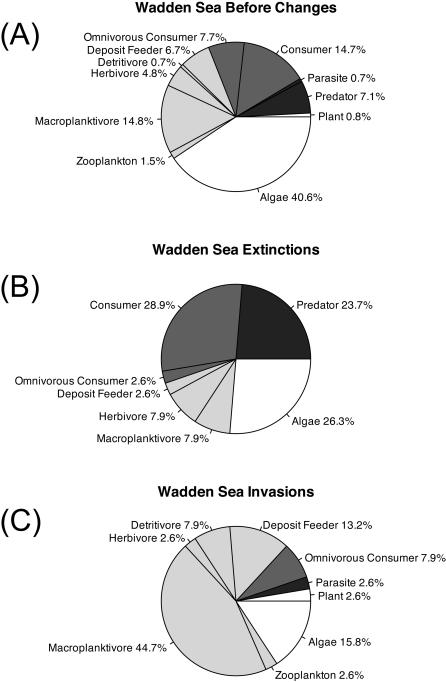
Trophic skew in the Wadden Sea in (A) uninvaded intact communities, (B) exotic species that have successfully established and (C) species that have gone extinct. Data presented as the percentage of species in each trophic group. Colors indicate trophic level (white = 1, light grey = 2, dark grey = 3, black = 4). Patterns match those in the larger global/regional data set (Fig. 2), with extinctions and invasions occurring within different trophic groups, and neither matching the natural trophic distribution of species.

**Figure 4:**
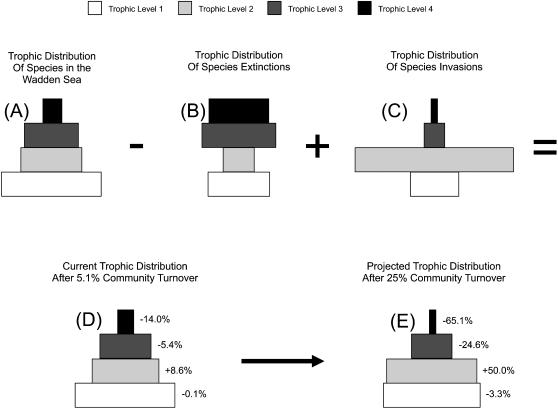
Trophic distribution of species in the Wadden Sea represented as trophic pyramids. Bar widths are scaled to the proportion of species in each trophic level and numbers indicate percentage of species loss or gain in each trophic level. Colors indicate trophic level (white = 1, light grey = 2, dark grey = 3, black = 4). (A) Pre-Disturbance (B) Skew of extinctions (C) Skew of Invasions (D) Current distribution after invasions and extinctions resulting in 5.1% community turnover (i.e., equal numbers of extinctions and invasions) (E) Projected change in the trophic distribution of species in the Wadden Sea for 25% community turnover. Note the dramatic reduction of the proportion of species in the top two trophic levels, and the increasing dominance of primary consumers.

These patterns of trophic skew from invasions and extinctions remain intact when analyses are restricted to spatially congruent subsets of the data for the Wadden Sea (Fig. 3, χ^2^_10_ = 41.47 p<0.0001). Both invasions and extinctions in the Wadden Sea differ in their pattern of species distributions among trophic levels from the trophic distribution of our reconstructed pre-disturbance species list (Fig. 3, Invasions χ^2^_10_ = 24.79 p = 0.0058, Extinctions χ^2^_10_ = 57.93 p<0.0001). The pre-invasion and extinction species list shows a classic “pyramid” in shape with decreasing numbers of species with increasing trophic level (Fig. 4A). The differential distribution of invasions and extinctions among trophic groups have already caused measurable changes in the relative distribution of species among trophic levels, even with invasions and extinctions each comprising only 5.1% of the total species (Fig 4D). While species richness has remained nearly the same, there are now 14% fewer predator species and 8.6% more primary consumer species.

These results suggest that marine ecosystems have already experienced a shift in food web architecture, with shrinking numbers of predatory species being replaced by an increasing diversity of suspension and deposit feeders. More extreme shifts will likely result from continued species invasions and extinction (Fig. 4E). For example, when the number of invasions and extinctions equals 25% of the total number of species, predator diversity will decline by 65% while primary consumer diversity will increase by 50%. Although this level of community turnover may seem extreme, some taxa such as birds and plants on some island ecosystems have already experienced similar or greater changes [18].

## Discussion

Our results show that, for the coastal marine systems we examined, extinctions are reducing the number of predatory species and secondary consumers while invasions are greatly increasing the number of primary filter feeders, detritivores, deposit feeders, and other primary consumers. We suspect the bias of extinctions toward higher trophic levels and invasions toward lower trophic levels will be consistent in other systems, although subtle differences may occur. For example, the influence of invasions in altering trophic skew may be reduced in open coast or oceanic environments relative to estuaries [19], although the loss of top predators appears to be a global phenomenon. Marine and terrestrial systems also likely differ in the trophic distribution of invaders due to differences in the relative importance of different vectors of introduction. In marine systems there are surprisingly few plant or macroalgal invasions relative to the large number documented on land [e.g., 18], while the numerically most abundant group of marine invaders, sessile planktivores, are virtually absent in terrestrial systems. Thus while heavily invaded coastal marine ecosystems experience a net increase in primary consumer richness, terrestrial systems may experience greater species gain at the producer level.

Research on the consequences of these types of changes in the number of species at multiple trophic levels is still relatively new [11], despite a thorough understanding of the consequences of changing the numbers of individuals at different trophic levels [20]. Some recent mesocosm and laboratory experiments with simplified communities suggest that, in both marine and terrestrial communities, loss of predator species alone may enhance the abundance and diversity of species at lower trophic levels even without invasions [11], [21]–[25]. While these results are largely from controlled experiments that may only include relatively strongly interacting species, they appear to be robust and match patterns observed from field surveys [22] and fisheries data [4] that incorporate total community richness or diversity. Additionally, with increasing deletions of large numbers of predator species and additions of planktivores, the likelihood of gaining or losing species with a particularly strong effect on ecosystem function increases. Indeed, both invasions and extinctions of strong interactors have been documented [e.g., 26], [27]. Even if many of the species on our lists of invasions and extinctions are not strong interactors, adding weakly interacting species can still have strong impacts on ecosystem stability [28] and have episodic strong effects with long lasting consequences [29].

Several studies also illustrate the difficulty of predicting the consequences of changing diversity at one trophic level without considering diversity at other trophic levels [30], [31]. For example, decreased predator diversity in California kelp forests is associated with increases in the abundance of herbivores and a concomitant reduction in kelp abundance [22]. This effect is caused by complementary responses of different species of herbivores to different species of predators, and therefore would not occur in systems with low herbivore diversity. Reductions in consumer diversity in a variety of eelgrass experiments have consistently resulted in enhanced epiphyte growth and reductions in sediment organic matter [32], [33], but the effects of herbivore diversity are strongest in the presence of predators [11].

A recent example from the Gulf of Maine is illustrative of the potential consequences of this multi-trophic phenomenon [34], [35]. Within the Gulf, overfishing has removed predatory fishes like cod, allowing native herbivore populations (particularly urchins) to increase, leading to a decline in native kelp. This decline has been exacerbated by two invasions at lower trophic levels: (1) an epiphytic bryozoan (*Membranipora membranacea*) that makes kelp brittle and more palatable to native grazers and (2) a grazer resistant alga, Dead Man's Fingers (*Codium fragile*), that fills gaps in the canopy and inhibits kelp recruitment. This fundamental shift in the subtidal landscape due to the synergistic effects of local extinctions of top predators and invasions of producers and suspension feeders has caused a major change in the structure of communities, including reductions in the recruitment of some native fish [34].

Given that the opposing patterns of trophic skew in extinctions and invasions we identify are consistent across a number of locations (Fig. 2 and 3), complex changes in trophic function are likely to be increasingly common. However, the general consequences of these multitrophic shifts may be quite variable. A recent meta-analysis shows that exotic herbivores are better than natives at controlling native plants. Native herbivores, in contrast, are better than exotics at controlling exotic plants [36]. This is the opposite of the preference found in the Gulf of Maine example. If this conclusion is as robust for other consumers as it appears to be for herbivores, introduction of exotic herbivores and elimination of native predators as suggested by our data could have a synergistically negative effect on native plants. The contradictory outcomes in these examples highlight our current inability to make specific predictions about the effects of simultaneous changes in species composition at multiple trophic levels. We therefore urge additional focused investigation into the ecological consequences of simultaneous diversity change at multiple trophic levels within these and other highly impacted ecosystems.

## Methods

To determine whether, at regional scales, the structure of marine food webs is being altered, we examined lists of marine species extinctions and invasions, classified species by trophic level and feeding mode, and then compared the trophic skew of invasions against that of extinctions. For species extinctions, we used the list of documented local and global marine extinctions from Dulvy [13] (n = 133 species). Because the consequences of diversity loss on ecosystem functioning are felt at the ecosystem (regional) level, we considered both global and regional extinctions in our analysis. In other words, if a species has been driven extinct from a region, it no longer performs any role in the food web of that region and thus for the purposes of regional ecosystem functioning is extinct. Therefore, both scales of extinction were relevant, as we were interested in loss of function at the scale of organisms within a food web.

For species introductions, we looked at published lists for San Francisco Bay [14] (n = 166 species), Australia [15] (n = 153 species), the Wadden Sea [16] (n = 34 species) and a previously unpublished list for the Gulf of the Farallones National Marine Sanctuary in Northern California (n = 141 species). We were able to directly compare invasions and extinctions (n = 38 species) in the Wadden Sea where patterns of changing functional group abundance have been previously documented [12], [37] in order to assess whether broader patterns held when considering invasions and extinctions for the same region. We then compared these data sets with a reconstruction of the “pre-disturbance” list of species in the Wadden Sea as estimated by taking an extant species list [17], excluding any invasive species and adding regionally extinct ones (n = 716 species total). Meiofauna, protozoans, and phytoplankton were only included in a minority of species lists (i.e., the Wadden Sea invasions, Australian invasions, and the “natural” Wadden Sea lists). They were not included in any of the extinction lists, potentially due to taxonomic uncertainty or difficulty of detecting extinctions in these poorly known groups. We therefore excluded them from our analysis. We also limited our analysis to species that occurred in marine or estuarine environments. The data for each extinction and invasion list are presented in Supplementary Tables S1, S2, S3, S4, and S5 (accompanied by References S1).

For each list of species lost or gained, we classified species into twelve different groups based on their primary food source and mode of feeding (Fig. 1). We aggregated these groups into four trophic levels. Trophic level one consisted of primary producers: vascular plants, benthic and multicellular algae, and phytoplankton (excluded from analysis, see above). Trophic level two consisted of A) herbivores, which consume vascular plants and algae, B) deposit feeders, which consume both sediment organic matter and detritus encountered in the sediment, C) detritivores, which specialize on detrital matter, D) zooplankton, which are both planktonic themselves and consume both phytoplankton and other zooplankton, and E) macroplanktivores, which are either benthic or pelagic, consume phytoplankton and/or zooplankton, and are large enough not to be consumed by other planktivores. Trophic level three consisted of consumers, which eat one or more species in trophic level two, and may also consume other members of trophic level three. Trophic level three also contained omnivorous consumers, which eat species from both trophic level one and two, and may also eat other species in trophic level three. Trophic level four consisted of predators, which eat either consumers or omnivorous consumers, and parasites which utilize animals at all trophic levels.

We assigned species to trophic groups based on published literature. Plants and algae were classified without a reference. Cnidarians, sponges, ascidians, bryozoans, barnacles, and mussels were all classified as macroplanktivores based on their feeding biology. When the trophic group of a species could not be determined, we used information known about the trophic status of the lowest possible taxonomic group containing the species. In some cases, species fit in multiple trophic groups. As they added multiple functions to the food web, and we wished to look at changes in food web structure rather than total biodiversity *per se*, we included species in multiple categories for our functional group analyses. Species were only counted once when looking at change in number of species per trophic level. By design of our trophic groupings, no species fit in multiple trophic levels.

We recognize that classification of some feeding groups into particular trophic levels is not straightforward given widespread omnivory. In particular, many benthic and pelagic planktivorous organisms are facultatively if not obligately omnivorous and thus could be considered “omnivorous consumers” in trophic level three instead of in trophic level two where we placed them. As there were few zooplankton in our data set, we do not think that their classification in level two or three is likely to qualitatively alter the results. We retain the “macroplanktivores” which are mostly benthic suspension feeders in trophic level 2 rather than 3 because the types of organisms that consume them are similar to those that consume other members of trophic level two (herbivores, detritivores) and these predators largely reside themselves within trophic level three. In other words, had we moved macroplanktivores to trophic level 3, we would have then shifted the species at trophic level 3 and 4 to levels 4 and 5. Thus the placement of planktivores in trophic level 2 or 3 would not fundamentally alter our conclusions.

Trophic skew was assessed using contingency analysis in R to compare the number of species in each trophic group (both trophic level and functional feeding group) in the list of species extinctions to each list of species invasions. Results of both analyses are presented and do not qualitatively differ.

## Supporting Information

Table S1 Lists of marine species extinctions from Dulvy (2003), their trophic group, and reference for trophic group from literature survey. Reference list follows in supplementary references S1.
(0.32 MB DOC)

Table S2 Lists of marine species invasions in San Francisco Bay from Cohen and Carlton (1995), their trophic group, and reference for trophic group from literature survey. Reference list follows in supplementary references S1.
(0.37 MB DOC)

Table S3 List of marine species invasions in the Gulf of the Farallones National Marine Sanctuary, their trophic group, and reference for trophic group from literature survey. Reference list follows in supplementary references S1.
(0.34 MB DOC)

Table S4 List of marine species invasions in Australia from NIMPIS, their trophic group, and reference for trophic group from literature survey. Reference list follows in supplementary references S1.
(0.36 MB DOC)

Table S5 List of marine species invasions in the Wadden Sea from Nehring (2006), their trophic group, and reference for trophic group from literature survey. Reference list follows in supplementary references S1. For a full list of all species in the Wadden Sea classified in trophic groups, please contact the authors.
(0.11 MB DOC)

References S1 Reference list for trophic classification from literature survey of all species in supplementarytables S1, S2, S3, S4 and S5.
(0.08 MB DOC)
