# Supplementary material for: Invasions and Extinctions Reshape Coastal Marine Food Webs
Source: PLoS One. 2007 Mar 14;2(3):e295. doi: 10.1371/journal.pone.0000295 (PMC1808429; doi:10.1371/journal.pone.0000295)
Supplement: Table S1 — Lists of marine species extinctions from Dulvy (2003), their trophic group, and reference for trophic group from literature survey. Reference list follows in supplementary references S1. (0.32 MB DOC) [file pone.0000295.s001.doc]

# Supplementary Table S1

List of marine species extinctions from Dulvy (2003), their trophic group, and reference for trophic group from literature survey. Reference list follows in supplementary references S1.

| Table S1: Extinctions from Dulvy 2003 | |  |  |
| --- | --- | --- | --- |
| **Species Name** | **Common Name** | **Trophic Group** | **Reference** |
| *Antithamnion vollosum* | Red algae | algae |  |
| *Ceramium diaphanum* | Red algae | algae |  |
| *Cladophora dalmatica* | Green algae | algae |  |
| *Colpomenia peregrina* | Brown algae | algae |  |
| *Corallina officinalis* | Red algae | algae |  |
| *Gigartina australis* | Turkish towel algae | algae |  |
| *Jania rubens* | Red algae | algae |  |
| *Pneophyllum fragile* | Red algae | algae |  |
| *Punctaria hiemalis* | Brown algae | algae |  |
| *Spermothamnion repens* | Red algae | algae |  |
| *Sphacelaria cirrosa* | Brown algae | algae |  |
| *Vanvoorstia bennettiana* | Bennett's seaweed | algae |  |
| *Acipenser oxyrhinchus* *oxyrhinchus* | Atlantic sturgeon | consumer | [1] |
| *Acipenser sturio* | European sturgeon | consumer | [2] |
| *Acipenser sturio* | European sturgeon | consumer | [2] |
| *Alca impennis* | Great Auk | consumer | [3]; [4] |
| *Alosa alosa* | Allis shad | consumer | [2] |
| *Anampses viridis* | Green wrasse | consumer | [2] |
| *Antennarius pauciradiatus* | Dwarf frogfish | consumer | [2] |
| *Bahaba taipingensis* | Chinese bahaba | consumer | [2] |
| *Buccinum undatum* | Whelk | consumer | [5] |
| *Camptorhynchus labradorius* | Labrador duck | consumer | [6] |
| *Corambe obscura* | Obscure corambe | consumer | [5] |
| *Coregonus oxyrinchus* | Whitefish | consumer | [2] |
| *Cosmocampus brachycephalus* | Crested pipefish | consumer | [2] |
| *Ctenogobius boleosoma* | Darter goby | consumer | [2] |
| *Dipturus alba* | White skate | consumer | [2] |
| *Dipturus batis* | Common skate | consumer | [2] |
| *Dipturus batis* | Common skate | consumer | [2] |
| *Dipturus batis* | Common skate | consumer | [2] |
| *Dipturus oxyrhinchus* | Long-nose skate | consumer | [7] |
| *Dipturus oxyrhinchus* | Long-nose skate | consumer | [7] |
| *Eschrichtius robustus* | Gray whale | consumer | [6] |
| *Gillellus greyae* | Arrow stargazer | consumer | [2] |
| *Haematopus meadewaldoni* | Canary Islands oystercatcher | consumer | [8] |
| *Homarus gammarus* | Lobster | consumer | [9] |
| *Leucoraja circularis* | Sandy ray | consumer | [2] |
| *Leucoraja circularis* | Sandy ray | consumer | [2] |
| *Leucoraja mirelatus* | Maltese ray | consumer | [2] |
| *Leucoraja naevis* | Cuckoo ray | consumer | [2] |
| *Mergus australis* | Auckland Islands merganser | consumer | [6] |
| *Mustelus asterias* | Starry smoothhound | consumer | [2] |
| *Myliobatis aquila* | Common eagle ray | consumer | [2] |
| *Mysidopsis angusta* |  | consumer | [9] |
| *Oxymonacanthus longirostris* | Harlequin leatherjacket | consumer | [2] |
| *Oxynotus centrina* | Angular rough shark | consumer | [2] |
| *Oxynotus centrina* | Angular rough shark | consumer | [2] |
| *Paraclinus nigripinnis* | Blackfin blenny | consumer | [2] |
| *Pelecanus crispus* | Dalmatian pelican | consumer | [10] |
| *Pristis perotteti* | Largetooth sawfish | consumer | [2] |
| *Prototoctes oxyrhynchus* | New Zealand grayling | consumer | [2] |
| *Raja clavata* | Thornback ray | consumer | [2] |
| *Salmo salar* | Atlantic salmon | consumer | [2] |
| *Salmo salar* | Atlantic salmon | consumer | [2] |
| *Scorpaena grandicornis* | Plumed scorpionfish | consumer | [2] |
| *Scyliorhinus canicula* | Small-spotted catshark or lesser spotted dogfish | consumer | [2] |
| *Somateria mollissima* | Eider duck | consumer | [11] |
| *Spinachia spinachia* | Fifteen-spined stickleback | consumer | [2] |
| *Sterna dougallii* | Roseate tern | consumer | [12] |
| *Syngnathus typhle* | Deep-snouted pipefish | consumer | [2] |
| *Trachinus draco* | Greater weaver | consumer | [2] |
| *Apogon affinis* | Bigtooth cardinalfish | consumer omnivore | [2] |
| *Apogon robinsi* | Roughlip cardinalfish | consumer omnivore | [2] |
| *Balaena mysticetus* | Bowhead Whale | consumer omnivore | [13] |
| *Bolbometopon muricatum* | Bumphead parrotfish | consumer omnivore | [2] |
| *Cerithidea californica* | Mudsnail | consumer omnivore | [14]; [5] |
| *Cerithidea fuscata* | Horn snail | consumer omnivore | [15] |
| *Dugong dugon* | Dugong | consumer omnivore | [6] |
| *Lactophrys trigonus* | Buffalo trunkfish | consumer omnivore | [2] |
| *Phaeoptyx conklini* | Freckled cardinalfish | consumer omnivore | [2] |
| *Phyllaplysia smaragda* | Emerald leafslug | consumer omnivore | [5] |
| *Rhithropanopeus harrisii* | Harris mud crab | consumer omnivore | [16] |
| *Alkmaria romijni* |  | deposit feeder | [17] |
| *"Collisella" edmitchelli* | Rocky shore limpet | herbivore | [5] |
| *Diadema antillarum* | Long-spined urchin | herbivore | [18] |
| *Haliotus sorenseni* | White abalone | herbivore | [5] |
| *Hydrodamalis gigas* | Steller's sea cow | herbivore | [6] |
| *Lacuna vincta* | Northern lacuna | herbivore | [15] |
| *Littoraria flammea* | Periwinkle | herbivore | [5] |
| *Lottia alveus alveus* | Atlantic eelgrass limpet | herbivore | [19] |
| *Nicholsina usta* | Emerald parrotfish | herbivore | [2] |
| *Onoba [Cingula] semicostata aculeus* |  | herbivore | [20] |
| *Paracentrotus lividus* | Purple sea urchin | herbivore | [18] |
| *Rissoa membranacea* |  | herbivore | [21] |
| *Stiliger vossi* | Stiliger vossi | herbivore | [5] |
| *Tripneustes gratilla* | Short-spined sea urchin | herbivore | [22] |
| *Acanthomysis longicornis* | mysid | macroplanktivore | [23] |
| *Azurina eupalama* | Galapagos damsel | macroplanktivore | [2] |
| *Clupea harengus* | Herring (Zuiderzee race) | macroplanktivore | [2] |
| *Clupea harengus* | Icelandic spring-spawning population | macroplanktivore | [2] |
| *Crassostrea virginica* | Eastern oyster | macroplanktivore | [15] |
| *Edwardsia ivella* | anemone | macroplanktivore |  |
| *Eubalaena glacialis* | Right whale | macroplanktivore | [6] |
| *Hippopus hippopus* | Giant clam | macroplanktivore | [9] |
| *Millepora boschmai* | Fire coral | macroplanktivore |  |
| *Mytilus trossulus* | Foolish mussel | macroplanktivore | [15] |
| *Ostrea edulis* | Edible oyster | macroplanktivore | [5] |
| *Sabellaria spinulosa* |  | macroplanktivore | [24] |
| *Siderastrea glynni* | stony coral | macroplanktivore | [25] |
| *Tridacna gigas* | Giant clam | macroplanktivore | [9] |
| *Upogebia bermudensis* | mud shrimp | macroplanktivore | [5] |
| *Argyrosomus regius* | Meagre | predator | [2] |
| *Dasyatis pastinaca* | Stingray | predator | [2] |
| *Echinorhinus brucus* | Bramble shark | predator | [2] |
| *Enchelycore anatina* | Fangtooth moray | predator | [2] |
| *Enhydra lutris* | Sea otter | predator | [6] |
| *Epinephelus marginatus* | Dusky grouper | predator | [2] |
| *Galeorhinus galeus* | Tope or soupfin shark | predator | [2] |
| *Galeorhinus galeus* | Tope or soupfin shark | predator | [2] |
| *Haliaeetus albicilla* | White-tailed eagle | predator | [10] |
| *Halichoerus grypus* | Grey seal | predator | [6] |
| *Heptranchias perlo* | Sharpnose seven gill shark | predator | [2] |
| *Hydroprogne caspia* | Caspian tern | predator | [26] |
| *Larus canus* | Common gull | predator | [6] |
| *Larus fuscus* | black-backed gull | predator | [27] |
| *Monachus tropicalis* | West Indian monk seal | predator | [6] |
| *Mustela macrodon* | Sea mink | predator | [28] |
| *Mustelus mustelus* | Smoothhound | predator | [2] |
| *Mustelus mustelus* | Smoothhound | predator | [2] |
| *Phalacrocorax perspicillatus* | Pallas's cormorant | predator | [10] |
| *Phoca vitulina* | Harbour seal | predator | [6] |
| *Phocoena phocoena* | Harbour porpoise | predator | [6] |
| *Phocoena phocoena* | Harbour porpoise | predator | [6] |
| *Pristis pectinata* | Smalltooth sawfish | predator | [2] |
| *Pristis pectinata* | Smalltooth sawfish | predator | [2] |
| *Raja microocellata* | Small-eyed ray | predator | [2] |
| *Rostroraja alba* | White skate | predator | [2] |
| *Scyliorhinus stellarus* | Nurse hound | predator | [2] |
| *Squatina squatina* | Angel shark | predator | [2] |
| *Squatina squatina* | Angel shark | predator | [2] |
| *Squatina squatina* | Angel shark | predator | [2] |
| *Tursiops truncatus* | Bottlenose dolphin | predator | [6] |
| *Tursiops truncatus* | Bottlenose dolphin | predator | [6] |
|  |  |  |  |
