# Supplementary material for: Invasions and Extinctions Reshape Coastal Marine Food Webs
Source: PLoS One. 2007 Mar 14;2(3):e295. doi: 10.1371/journal.pone.0000295 (PMC1808429; doi:10.1371/journal.pone.0000295)
Supplement: Table S2 — Lists of marine species invasions in San Francisco Bay from Cohen and Carlton (1995), their trophic group, and reference for trophic group from literature survey. Reference list follows in supplementary references S1. (0.37 MB DOC) [file pone.0000295.s002.doc]

# Supplementary Table S2

List of marine species invasions in San Francisco Bay from Cohen and Carlton (1995), their trophic group, and reference for trophic group from literature survey. Reference list follows in supplementary references S1.

| Table S2: Invasions in San Francisco Bay | |  |  |
| --- | --- | --- | --- |
| **Species Name** | **Taxonomic Group** | **Trophic Group** | **Reference** |
| *Bryopsis sp* | Algae | algae |  |
| *Callithamnion byssoides* | Algae | algae |  |
| *Codium fragile tomentisiudes* | Algae | algae |  |
| *Polysophonia denudata* | Algae | algae |  |
| *Sargassum muticum* | Algae | algae |  |
| *Alosa sapidissima* | Fish | consumer | [29] |
| *Anisolabis maritima* | Insecta | consumer | [29] |
| *Busycotypus canaliculatus* | Gastropoda | consumer | [30] |
| *Carcinus maenas* | Crustacea | consumer | [29] |
| *Catriona rickettsi* | Gastropoda | consumer | [29] |
| *Dorosoma petenense* | Fish | consumer | [29] |
| *Eubranchus misakiensis* | Gastropoda | consumer | [29] |
| *Marphysa sanguinea* | Annelida | consumer | [31] |
| *Okenia plana* | Gastropoda | consumer | [29] |
| *Palaemon macrodactylus* | Crustacea | consumer | [29] |
| *Philine auriformis* | Gastropoda | consumer | [29] |
| *Sakuraeolis enosimensis* | Gastropoda | consumer | [29] |
| *Snidotea laevidorsalis* | Crustacea | consumer | [29] |
| *Tenellia adspersa* | Gastropoda | consumer | [29] |
| *Urosalpinx cinerea* | Gastropoda | consumer | [29] |
| *Cyprinus carpio* | Fish | consumer omnivore | [29] |
| *Eriocheir sinensis* | Crustacea | consumer omnivore | [32] |
| *Gammarus daiberi* | Crustacea | consumer omnivore | [29] |
| *Ilyanassa obsoleta* | Gastropoda | consumer omnivore | [29] |
| *Leucothe sp.* | Crustacea | consumer omnivore | [29] |
| *Melita nitida* | Crustacea | consumer omnivore | [29] |
| *Melita sp.* | Crustacea | consumer omnivore | [29] |
| *Nereis succinea* | Annelida | consumer omnivore | [29] |
| *Pacifastacus leniusculus* | Crustacea | consumer omnivore | [32] |
| *Paradexamine sp.* | Crustacea | consumer omnivore | [29] |
| *Parapleustes derzhavini* | Crustacea | consumer omnivore | [29] |
| *Rhithropanopeus harrisii* | Crustacea | consumer omnivore | [33] |
| *Stenothe valida* | Crustacea | consumer omnivore | [29] |
| *Transorchestia enigmatica* | Crustacea | consumer omnivore | [29] |
| *Heteromastus filiformis* | Capitellid worm | deposit feeder | [34] |
| *Paranais frici* | Annelida | deposit feeder | [35] |
| *Polydora ligni* | Annelida | deposit feeder | [36] |
| *Potamothrix bavaricus* | Annelida | deposit feeder | [37] |
| *Sabaco elongatus* | Annelida | deposit feeder | [9] |
| *Eusarsiella zostericola* | Crustacea | deposit feeder, detritivore | [38] |
| *Limnodrilus monothecus* | Annelida | deposit feeder, detritivore | [29] |
| *Tubificoides apectinatus* | Annelida | deposit feeder, detritivore | [29] |
| *Tubificoides brownae* | Annelida | deposit feeder, detritivore | [29] |
| *Tubificoides wasselli* | Annelida | deposit feeder, detritivore | [29] |
| *Marenzelleria viridis* | Annelida | deposit feeder, detritivore, macroplanktivore | [39]; [40] |
| *Boccardiella ligerica* | Annelida | deposit feeder, macroplanktivore | [41] |
| *Chelura terebrans* | Crustacea | detritivore | [29] |
| *Eurylana arcuata* | Crustacea | detritivore | [42] |
| *Iais californica* | Crustacea | detritivore | [29] |
| *Limnoria quadripunctata* | Crustacea | detritivore | [29] |
| *Limnoria tripunctata* | Crustacea | detritivore | [29] |
| *Streblospio benedicti* | Annelida | detritivore | [34] |
| *Ampelisca abdita* | Crustacea | detritivore, macroplanktivore | [38] |
| *Teredo navalis* | Bivalvia | detritivore, macroplanktivore | [29] |
| *Littorina saxatilis* | Gastropoda | herbivore | [29] |
| *Ovatella myosotis* | Gastropoda | herbivore | [30] |
| *Procambarus clarkii* | Crustacea | herbivore | [29] |
| *Trigonotylus uhleri* | Insecta | herbivore | [29] |
| *Ampithoe valida* | Crustacea | herbivore, macroplanktivore, detritvore | [38] |
| *Vnerupis philippinarum* | Bivalvia | macropanktivore | [30] |
| *Alcyonidium polyoum* | Bryozoa | macroplanktivore | [29] |
| *Anguinella palmata* | Bryozoa | macroplanktivore | [29] |
| *Ascidia sp.* | Tunicata | macroplanktivore | [29] |
| *Aurelia "aurita"* | Cnidaria | macroplanktivore | [29] |
| *Balanus amphitrite* | Crustacea | macroplanktivore | [38] |
| *Balanus improvisus* | Crustacea | macroplanktivore | [43] |
| *Barentsia benedeni* | Entoprocta | macroplanktivore | [15] |
| *Blackfordia virginica* | Cnidaria | macroplanktivore | [38] |
| *Botryllus aurantius* | Tunicata | macroplanktivore | [29] |
| *Botryllus schlosseri* | Tunicata | macroplanktivore | [43] |
| *Botryllus sp.* | Tunicata | macroplanktivore | [29] |
| *Bowerbankia gracilis* | Bryozoa | macroplanktivore | [29] |
| *Bugula "neritina"* | Bryozoa | macroplanktivore | [29] |
| *Bugula stolonifera* | Bryozoa | macroplanktivore | [29] |
| *Ciona intestinalis* | Tunicata | macroplanktivore | [43] |
| *Ciona savignyi* | Tunicata | macroplanktivore | [43] |
| *Cipangopaludina chinensis malleata* | Gastropoda | macroplanktivore | [44] |
| *Cladonema uchidai* | Cnidaria | macroplanktivore | [29] |
| *Clava multicornis* | Cnidaria | macroplanktivore | [29] |
| *Cliona sp* | Porifera | macroplanktivore | [5] |
| *Conopeum tenuissimum* | Bryozoa | macroplanktivore | [29] |
| *Corbicula fluminea* | Bivalvia | macroplanktivore | [44] |
| *Cordylophora capia* | Cnidaria | macroplanktivore | [29] |
| *Corymorpha sp* | Cnidaria | macroplanktivore | [29] |
| *Crepidula plana* | Gastropoda | macroplanktivore | [32] |
| *Cryptosula pallasiana* | Bryozoa | macroplanktivore | [29] |
| *Diadumene ?cincta* | Cnidaria | macroplanktivore | [29] |
| *Diadumene leucolena* | Cnidaria | macroplanktivore | [29] |
| *Diadumene lineata* | Cnidaria | macroplanktivore | [29] |
| *Diadumne franciscana* | Cnidaria | macroplanktivore | [29] |
| *Epinebalia sp* | Crustacea | macroplanktivore | [38] |
| *Ficopomatus enigmaticus* | Annelida | macroplanktivore | [30] |
| *Garveia franciscana* | Cnidaria | macroplanktivore | [29] |
| *Gonothyraea clarki* | Cnidaria | macroplanktivore | [29] |
| *Halichondria bowerbanki* | Porifera | macroplanktivore | [29] |
| *Haliclona loosanoffi* | Porifera | macroplanktivore | [5] |
| *Jassa marmorata* | Crustacea | macroplanktivore | [38] |
| *Lyrodus pedicellatus* | Bivalvia | macroplanktivore | [45] |
| *Maeotias inexspectata* | Cnidaria | macroplanktivore | [29] |
| *Microciona prolifera* | Porifera | macroplanktivore | [29] |
| *Molgula manhattensis* | Tunicata | macroplanktivore | [29] |
| *Musculista senhousia* | Bivalvia | macroplanktivore | [29] |
| *Mya arenaria* | Bivalvia | macroplanktivore | [30] |
| *Mytilus galloprovincialis* | Bivalvia | macroplanktivore | [46] |
| *Obelia ?bidentata* | Cnidaria | macroplanktivore | [17] |
| *Obelia ?dichontoma* | Cnidaria | macroplanktivore | [29] |
| *Petricolaria pholadiformis* | Bivalvia | macroplanktivore | [29] |
| *Potamilla sp.* | Annelida | macroplanktivore | [5] |
| *Potamocorbula amurensis* | Bivalvia | macroplanktivore | [29] |
| *Prosuberites sp* | Porifera | macroplanktivore | [29] |
| *Sarsia tubulosa* | Cnidaria | macroplanktivore | [47] |
| *Schizoporella unicornis* | Bryozoa | macroplanktivore | [29] |
| *Styela clava* | Tunicata | macroplanktivore | [29] |
| *Theora fragilis* | Bivalvia | macroplanktivore | [29] |
| *Tubularia crocea* | Cnidaria | macroplanktivore | [29] |
| *Victorella pavida* | Bryozoa | macroplanktivore | [29] |
| *Watersipora "subtorquata"* | Bryozoa | macroplanktivore | [29] |
| *Zoobotryon verticillatum* | Bryozoa | macroplanktivore | [29] |
| *Arcuatula demissa* | Bivalvia | macroplanktivore, deposit feeder | [48] |
| *Corophinum heteroceratum* | Crustacea | macroplanktivore, deposit feeder | [29] |
| *Macoma petalum* | Bivalvia | macroplanktivore, deposit feeder | [29] |
| *Pseudopolydora kempi* | Annelida | macroplanktivore, deposit feeder | [49] |
| *Corophium acherusicum* | Crustacea | macroplanktivore, deposit feeder, detritivore | [43] |
| *Corophium alienense* | Crustacea | macroplanktivore, deposit feeder, detritivore | [43] |
| *Corophium insidiosum* | Crustacea | macroplanktivore, deposit feeder, detritivore | [43] |
| *Acanthomysis aspera* | Crustacea | macroplanktivore, detritivore | [43] |
| *Acanthomysis sp.* | Crustacea | macroplanktivore, detritivore | [15] |
| *Caprella mutica* | Crustacea | macroplanktivore, detritivore | [29] |
| *Crepidula convexa* | Gastropoda | macroplanktivore, detritivore | [32] |
| *Deltamysis holmquistae* | Crustacea | macroplanktivore, detritivore | [15] |
| *Dynoides dentisinus* | Crustacea | macroplanktivore, detritivore | [15] |
| *Gemma gemma* | Bivalvia | macroplanktivore, detritivore | [34] |
| *Grandidierella japonica* | Crustacea | macroplanktivore, detritivore | [43] |
| *Nippoleucon hinumensis* | Crustacea | macroplanktivore, detritivore | [15] |
| *Paranthura sp.* | Crustacea | macroplanktivore, detritivore | [15] |
| *Sinelobus sp.* | Crustacea | macroplanktivore, detritivore | [29] |
| *Sphaeroma quoyanum* | Crustacea | macroplanktivore, detritivore | [29] |
| *Pseudopolydora paucibranchiata* | Annelida | macroplantkivore, deposit feeder | [50] |
| *Boonea bisuturalis* | Gastropoda | parasite | [29] |
| *Mytilicola orientalis* | Crustacea | parasite | [29] |
| *Limnoithona tetraspina* | Crustacea | planktivore | [51] |
| *Oithona davisae* | Crustacea | planktivore | [43]; [52] |
| *Pseudodiaptomus forbesi* | Crustacea | planktivore | [53] |
| *Pseudodiaptomus marinus* | Crustacea | planktivore | [29] |
| *Sinocalanus doerrii* | Crustacea | planktivore | [29] |
| *Tortanus sp.* | Crustacea | planktivore | [29] |
| *Acartiella sinensis* | Crustacea | planktivore, detritivore | [29] |
| *Limnoithona sinensis* | Crustacea | planktivore, detritivore | [54] |
| *Chenopodium macrospermum var halophium* | Vascular Plant | plant |  |
| *Cotula coronopifolia* | Vascular Plant | plant |  |
| *Eichhornia crassipes* | Vascular Plant | plant |  |
| *Limosella subulata* | Vascular Plant | plant |  |
| *Polygonum patulum* | Vascular Plant | plant |  |
| *Polypogon elongatus* | Vascular Plant | plant |  |
| *Salsola soda* | Vascular Plant | plant |  |
| *Spartina alterniflora* | Vascular Plant | plant |  |
| *Spartina anglica* | Vascular Plant | plant |  |
| *Spartina densiflora* | Vascular Plant | plant |  |
| *Spartina patens* | Vascular Plant | plant |  |
| *Spergularia media* | Vascular Plant | plant |  |
| *Acanthogobius flavimanus* | Fish | predator | [55] |
| *Gambusia affinis* | Fish | predator | [29] |
| *Lucania parva* | Fish | predator | [56] |
| *Ondatra Zibethicus* | mammal | predator | [29] |
| *Tridentiger bifasciatus* | Fish | predator | [29] |
| *Tridentiger trigonocephalus* | Fish | predator | [55] |
|  |  |  |  |
