# Supplementary material for: Invasions and Extinctions Reshape Coastal Marine Food Webs
Source: PLoS One. 2007 Mar 14;2(3):e295. doi: 10.1371/journal.pone.0000295 (PMC1808429; doi:10.1371/journal.pone.0000295)
Supplement: Table S3 — List of marine species invasions in the Gulf of the Farallones National Marine Sanctuary, their trophic group, and reference for trophic group from literature survey. Reference list follows in supplementary references S1. (0.34 MB DOC) [file pone.0000295.s003.doc]

# Supplementary Table S3

Lists of marine species invasions in the Gulf of the Farallones National Marine Sanctuary, their trophic group, and reference for trophic group from literature survey. Reference list follows in supplementary references S1.

| **Table S3: Invasions in the Gulf of the Farallones National Marine Sanctuary** | | | |  |
| --- | --- | --- | --- | --- |
| **Species Name** | **Common Name** | **Trophic Group** | **Reference** | |
| *Aglaothamnion cordatum* | Rhodophycota | algae |  | |
| *Codium fragile tomentosoides* | Dead Man's Fingers | algae |  | |
| *Gelidium vagum* | red algae | algae |  | |
| *Polysiphonia denudata* | Red Siphonweed | algae |  | |
| *Sargassum muticum* | British Wireweed | algae |  | |
| *Undaria pinnatifida* | Wakame | algae | [15] | |
| *Acanthogobius flavimanus* | Yellowfin goby | consumer | [2] | |
| *Alosa sapidissima* | Atlantic Shad | consumer | [29] | |
| *Carcinus maenas* | Green Crab | consumer | [29] | |
| *Ceratostoma inornatum* | Japanese oyster drill | consumer | [5] | |
| *Exogone lourei* | polychaete | consumer | [9] | |
| *Glycera americana* | polychate | consumer | [57] | |
| *Harmothoe imbricata* | polychate | consumer | [31] | |
| *Okenia plana* | Flat Okenia (nudibranch) | consumer | [58] | |
| *Palaemon macrodactylus* | Korean Shrimp | consumer | [29] | |
| *Philine auriformis* | nudibranch | consumer | [29] | |
| *Philine orientalis* | nudibranch | consumer | [59] | |
| *Tenellia adspersa* | Miniature aeolis (nudibranch) | consumer | [29] | |
| *Urosalpinx cinerea* | Atlantic Oyster Drill | consumer | [29] | |
| *Caprella californica* | amphipod | consumer omnivore | [5] | |
| *Jassa slatteryi* | amphipod | consumer omnivore | [60] | |
| *Melita nitida* | amphipod | consumer omnivore | [29] | |
| *Neanthes succinea* | pileworm | consumer omnivore | [38] | |
| *Parapleustes derzhavini* | amphipod | consumer omnivore | [29] | |
| *Nassarius obsoletus* | Easterm Mud Snail | consumer omnivore, detritivore | [61]; [62] | |
| *Notomastus hemipodus* | polychate | consumer omnivore, detritivore | [63] | |
| *Cyprinus carpio* | European Carp | consumer, deposit feeder | [29] | |
| *Apoprionospio pygmaea* | spionid | deposit feeder | [15] | |
| *Capitella capitata Complex* | Bristleworm | deposit feeder | [5] | |
| *Ctenodrilus serratus* | teribellid polychaete | deposit feeder | [15] | |
| *Dipolydora socialis* | spionid polychaete | deposit feeder | [15] | |
| *Euchone limnicola* | fan worm polychate | deposit feeder | [38] | |
| *Heteromastus filiformis* | polychate | deposit feeder | [34] | |
| *Leptochelia dubia* | tanaid | deposit feeder | [15] | |
| *Mediomastus ambiseta* | polychate | deposit feeder | [64] | |
| *Polydora ligni* | Mud Worm | deposit feeder | [36] | |
| *Pseudopolydora paucibranchiata* | spionid | deposit feeder | [43] | |
| *Cumella vulgaris* | cumacean | deposit feeder, maroplanktivore | [9] | |
| *Ampithoe lacertosa* | amphipod | detritivore | [65] | |
| *Iais californica* | isopod | detritivore | [29] | |
| *Limnoria quadripunctata* | isopod | detritivore | [29] | |
| *Limnoria tripunctata* | isopod | detritivore | [29] | |
| *Streblospio benedicti* | spionid | detritivore | [15] | |
| *Ampelisca abdita* | amphipod | detritivore, macroplanktivore | [38] | |
| *Ampelisca agassizi* | amphipod | detritivore, macroplanktivore | [48] | |
| *Caprella mutica* | Skeleton Shrimp (amphipod) | detritivore, macroplanktivore | [29] | |
| *Nippoleucon hinumensis* | Asian cumacean | detritivore, macroplanktivore | [15] | |
| *Caprella acanthogaster* | amphipod | herbivore | [66] | |
| *Corophium uenoi* | amphipod | herbivore | [67] | |
| *Ianiropsis tridens* | isopod | herbivore | [9] | |
| *Jassa carltoni* | amphipod | herbivore | [43] | |
| *Myosotella myosotis* | European Melampus | herbivore | [30] | |
| *Paranthura elegans* | isopod | herbivore | [15] | |
| *Platynereis bicanaliculata* | polychate | herbivore | [60] | |
| *Sinocorophium alienense* | amphipod | herbivore | [15] | |
| *Sinocorophium heteroceratum* | amphipod | herbivore | [15] | |
| *Batillaria attramentaria* | Japanese False Cerith | herbivore, deposit feeder | [68] | |
| *Ampithoe valida* | amphipod | herbivore, macroplanktivore, detritvore | [38] | |
| *Venerupis philippinarum* | Japanese Littleneck Clam | macropanktivore | [30] | |
| *Acanthomysis bowmani* | mysid | macroplanktivore |  | |
| *Alcyonidium gelatinosum* | bryozoan | macroplanktivore | [15] | |
| *Alcyonidium parasiticum* | bryozoan | macroplanktivore | [15] | |
| *Alcyonidium polyoum* | bryozoan | macroplanktivore | [29] | |
| *Amathia vidovici* | bryozoan | macroplanktivore | [69] | |
| *Amphinema sp.* | hydrozoan | macroplanktivore | [70] | |
| *Ascidia zara* | ascidian | macroplanktivore | [15] | |
| *Aurelia aurita* | Moon Jelly | macroplanktivore | [29] | |
| *Balanus amphitrite* | barnacle | macroplanktivore | [43] | |
| *Balanus improvisus* | barnacle | macroplanktivore | [43] | |
| *Barentsia benedeni* | bryozoan | macroplanktivore | [15] | |
| *Botrylloides perspicuum* | tunicate | macroplanktivore | [30] | |
| *Botrylloides violaceus* | tunicate | macroplanktivore | [30] | |
| *Botryllus schlosseri* | tunicate | macroplanktivore | [5] | |
| *Bowerbankia gracilis* | bryozoan | macroplanktivore | [29] | |
| *Bugula neritina* | bryozoan | macroplanktivore | [29] | |
| *Bugula stolonifera* | bryozoan | macroplanktivore | [29] | |
| *Ciona intestinalis* | tunicate | macroplanktivore | [5] | |
| *Ciona savignyi* | tunicate | macroplanktivore | [43] | |
| *Cliona celata* | sponge | macroplanktivore | [5] | |
| *Cliona lobata* | sponge | macroplanktivore | [5] | |
| *Conopeum tenuissimum* | bryozoan | macroplanktivore | [29] | |
| *Cordylophora caspia* | hydrozoan | macroplanktivore | [29] | |
| *Crassostrea gigas* | Pacific Giant Oyster | macroplanktivore | [5] | |
| *Cryptosula pallasiana* | bryozoan | macroplanktivore | [29] | |
| *Diadumene franciscana* | San Francisco Anemone | macroplanktivore | [29] | |
| *Diadumene leucolena* | White Anemone | macroplanktivore | [29] | |
| *Didemnum sp.* | tunicate | macroplanktivore | [30] | |
| *Diplosoma listerianum* | tunicate | macroplanktivore | [38] | |
| *Ericthonius brasiliensis* | amphipod | macroplanktivore | [71] | |
| *Ficopomatus enigmaticus* | Tube Worm | macroplanktivore | [30] | |
| *Halichondria bowerbanki* | sponge | macroplanktivore | [29] | |
| *Haliclona loosanoffi* | sponge | macroplanktivore | [5] | |
| *Haliplanella lineata* | anemone | macroplanktivore | [31] | |
| *Hymeniacidon sinapium* | sponge | macroplanktivore | [9] | |
| *Jassa marmorata* | amphipod | macroplanktivore | [15] | |
| *Laticorophium baconi* | amphipod | macroplanktivore | [38] | |
| *Leucothoe alata* | amphipod | macroplanktivore | [72] | |
| *Lyrodus pedicellatus* | Blacktip Shipworm (mollusc) | macroplanktivore | [45] | |
| *Macoma balthica* | Baltic Macoma | macroplanktivore | [73] | |
| *Mercenaria mercenaria* | Northern Quahog | macroplanktivore | [16] | |
| *Molgula manhattensis* | tunicate | macroplanktivore | [29] | |
| *Monocorophium acherusicum* | amphipod | macroplanktivore | [38] | |
| *Monocorophium insidiosum* | amphipod | macroplanktivore | [38] | |
| *Monocorophium uenoi* | amphipod | macroplanktivore | [38] | |
| *Musculista senhousia* | Green Mussel | macroplanktivore | [29] | |
| *Mya arenaria* | Softshell Clam | macroplanktivore | [30] | |
| *Mytilus galloprovincialis* | Mediterranean mussel | macroplanktivore | [46] | |
| *Nutallia nutallia* | Mahogany Clam | macroplanktivore | [15] | |
| *Nuttallia obscurata* | Purple-Mahogany Clam | macroplanktivore | [15] | |
| *Obelia bidentata* | Doubletoothed Hydroid | macroplanktivore | [17] | |
| *Obelia dichotoma* | Sea Thread Hydroid | macroplanktivore | [29] | |
| *Ostrea edulis* | Edible oyster | macroplanktivore | [15] | |
| *Ostrea lurida* | Olympia Oyster | macroplanktivore | [15] | |
| *Polyandrocarpa zorritensis* | tunicate | macroplanktivore | [15] | |
| *Prosuberites sp.* | sponge | macroplanktivore | [29] | |
| *Pteria sterna* | Wing Oyster | macroplanktivore | [15] | |
| *Sarsia tubulosa* | Clapper Hydromedusa | macroplanktivore | [47] | |
| *Schizoporella unicornis* | Single Horn Bryozoan | macroplanktivore | [29] | |
| *Styela clava* | tunicate | macroplanktivore | [29] | |
| *Theora lubrica* | Asian semele | macroplanktivore | [43] | |
| *Trochammina hadai* | foraminifera | macroplanktivore | [15] | |
| *Tubularia crocea* | hydrozoan | macroplanktivore | [29] | |
| *Victorella pavida* | bryozoan | macroplanktivore | [29] | |
| *Watersipora subtorquata* | bryozoan | macroplanktivore | [29] | |
| *Pseudopolydora kempi* | spionid | macroplanktivore, deposit feeder | [74] | |
| *Corophium acherusicum* | amphipod | macroplanktivore, deposit feeder, detritivore | [43] | |
| *Corophium alienense* | amphipod | macroplanktivore, deposit feeder, detritivore | [43] | |
| *Corophium insidiosum* | amphipod | macroplanktivore, deposit feeder, detritivore | [43] | |
| *Acanthomysis aspera* | mysid | macroplanktivore, detritivore | [15] | |
| *Gemma gemma* | Amethyst Gemclam | macroplanktivore, detritivore | [34] | |
| *Grandidierella japonica* | amphipod | macroplanktivore, detritivore | [43] | |
| *Sinelobus sp.* | tanaid | macroplanktivore, detritivore | [29] | |
| *Sphaeroma quoyanum* | Sphaeromatid Isopod | macroplanktivore, detritivore | [29] | |
| *Cercaria batillariae* | Trematode | parasite | [75] | |
| *Mytilicola orientalis* | Red Worm (copepod) | parasite | [29] | |
| *Pseudodiaptomus marinus* | copepod | planktivore | [36] | |
| *Cakile maritima* | European Sea Rocket | plant |  | |
| *Cotula coronopifola* | Brassbuttons | plant |  | |
| *Salsola soda* | Russian Thistle | plant |  | |
| *Gambusia affinis* | Mosquitofish | predator | [30] | |
| *Lucania parva* | Rainwater Killifish | predator | [2] | |
| *Morone saxatilis* | Striped Bass | predator | [2] | |
|  |  |  |  | |
