# Supplementary material for: Invasions and Extinctions Reshape Coastal Marine Food Webs
Source: PLoS One. 2007 Mar 14;2(3):e295. doi: 10.1371/journal.pone.0000295 (PMC1808429; doi:10.1371/journal.pone.0000295)
Supplement: Table S4 — List of marine species invasions in Australia from NIMPIS, their trophic group, and reference for trophic group from literature survey. Reference list follows in supplementary references S1. (0.36 MB DOC) [file pone.0000295.s004.doc]

# Supplementary Table S4

List of marine species invasions in Australia from NIMPIS, their trophic group, and reference for trophic group from literature survey. Reference list follows in supplementary references S1.

| **Table S4: Invasions in Australia** |  |  |  |
| --- | --- | --- | --- |
| **Species Name** | **Common Name** | **Trophic Group** | **Reference** |
| *Antithamnion cruciatum* | Red macroalga | algae |  |
| *Antithamnionella spirographidis* | Red macroalga | algae |  |
| *Arthrocladia villosa* | Brown macroalga | algae |  |
| *Asperococcus compressus* | Brown macroalga | algae |  |
| *Botrytella micromora* | Brown macroalga | algae |  |
| *Chondria arcuata* | Red macroalga | algae |  |
| *Cladophora prolifera* | Common pincushion algae | algae |  |
| [*Codium fragile ssp tomentosoides*](http://www.marine.csiro.au/crimp/nimpis/spSummary.asp?txa=6916) | Dead man's fingers | algae |  |
| *Cottoniella fusiformis* |  | algae |  |
| *Deucalion levringii* | Red macroalga | algae |  |
| *Elachista orbicularis* | Brown macroalga | algae |  |
| *Gymnogongrus crenulatus* | Red macroalga | algae |  |
| *Medeiothamnion lyallii* | Red macroalga | algae |  |
| [*Polysiphonia brodiei*](http://www.marine.csiro.au/crimp/nimpis/spSummary.asp?txa=10040) | Red macroalga | algae |  |
| *Polysiphonia senticulosa* | Red macroalga | algae |  |
| *Schottera nicaeensis* | Red macroalga | algae |  |
| *Solieria filiformis* | Red macroalga | algae |  |
| *Sphacella subtilissima* |  | algae |  |
| *Stictyosiphon soriferus* | Brown macroalga | algae |  |
| *Striaria attenuata* | Brown macroalga | algae |  |
| [*Undaria pinnatifida*](http://www.marine.csiro.au/crimp/nimpis/spSummary.asp?txa=6808) | Wakame (Japanese) | algae |  |
| *Vaucheria piloboloides* |  | algae |  |
| [*Acanthogobius flavimanus*](http://www.marine.csiro.au/crimp/nimpis/spSummary.asp?txa=7175) | Yellowfin goby | consumer | [43] |
| [*Acentrogobius pflaumi*](http://www.marine.csiro.au/crimp/nimpis/spSummary.asp?txa=7186) | Streaked goby | consumer | [43] |
| *Cabestana cutacea africana* | gastropod | consumer | [9] |
| *Charybdis japonica* | Lady crab | consumer | [76] |
| *Eubranchus inabai* | a nudibranch | consumer | [58] |
| *Filellum serpens* | a hydrozoa | consumer |  |
| [*Forsterygion lapillum*](http://www.marine.csiro.au/crimp/nimpis/spSummary.asp?txa=7187) | Common triplefin | consumer | [43] |
| [*Forsterygion varium*](http://www.marine.csiro.au/crimp/nimpis/spSummary.asp?txa=7180) | Variable triplefin | consumer | [43] |
| *Gambusia holbrooki* | eastern mosquitofish | consumer | [77] |
| [*Godiva quadricolor*](http://www.marine.csiro.au/crimp/nimpis/spSummary.asp?txa=9917) | Sea slug | consumer | [43] |
| *Grahamina gymnota* | Tasmanian robust tripplefin fish | consumer | [2] |
| *Lateolabrax japonicus* | Japanese sea bass | consumer | [2] |
| *Palaemon macrodactylus* | Oriental shrimp | consumer | [78] |
| [*Polycera capensis*](http://www.marine.csiro.au/crimp/nimpis/spSummary.asp?txa=6157) | Conspicuous polycera | consumer | [43] |
| [*Polycera hedgpethi*](http://www.marine.csiro.au/crimp/nimpis/spSummary.asp?txa=6158) | Hedgpeth's dorid | consumer | [43] |
| [*Thecacera pennigera*](http://www.marine.csiro.au/crimp/nimpis/spSummary.asp?txa=6159) | Winged thecacera | consumer | [43] |
| [*Theora lubrica*](http://www.marine.csiro.au/crimp/nimpis/spSummary.asp?txa=6148) | East Asian bivalve | consumer | [43] |
| [*Tridentiger trigonocephalus*](http://www.marine.csiro.au/crimp/nimpis/spSummary.asp?txa=7177) | Chameleon goby | consumer | [43] |
| [*Alitta succinea*](http://www.marine.csiro.au/crimp/nimpis/spSummary.asp?txa=6464) | Pileworm | consumer omnivore | [43] |
| [*Asterias amurensis*](http://www.marine.csiro.au/crimp/nimpis/spSummary.asp?txa=6131) | Northern Pacific seastar | consumer omnivore | [43] |
| [*Astrostole scaber*](http://www.marine.csiro.au/crimp/nimpis/spSummary.asp?txa=6132) | Rough seastar | consumer omnivore | [43] |
| *Bullia annulata* | South African annulate Bullia | consumer omnivore | [9] |
| [*Carcinus maenas*](http://www.marine.csiro.au/crimp/nimpis/spSummary.asp?txa=6275) | European shore crab | consumer omnivore | [43] |
| [*Chiton glaucus*](http://www.marine.csiro.au/crimp/nimpis/spSummary.asp?txa=6151) | New Zealand chiton | consumer omnivore | [43] |
| [*Cirolana harfordi*](http://www.marine.csiro.au/crimp/nimpis/spSummary.asp?txa=6280) | Speckled pill bug | consumer omnivore | [43] |
| *Elasmopus rapax* | an amphipod | consumer omnivore | [79] |
| *Gammarus tigrinus* | Amphipod | consumer omnivore | [80] |
| [*Metacarcinus novaezelandiae*](http://www.marine.csiro.au/crimp/nimpis/spSummary.asp?txa=6274) | Pie-crust crab | consumer omnivore | [43] |
| *Oreochromis mossambicus* | Mozambique tilapia | consumer omnivore | [2] |
| [*Patiriella regularis*](http://www.marine.csiro.au/crimp/nimpis/spSummary.asp?txa=6133) | New Zealand seastar | consumer omnivore | [43] |
| [*Pseudopolydora paucibranchiata*](http://www.marine.csiro.au/crimp/nimpis/spSummary.asp?txa=6128) | Japanese polydorid | consumer omnivore | [43] |
| [*Pyromaia tuberculata*](http://www.marine.csiro.au/crimp/nimpis/spSummary.asp?txa=6278) | Fire crab | consumer omnivore | [43] |
| [*Sphaeroma walkeri*](http://www.marine.csiro.au/crimp/nimpis/spSummary.asp?txa=8598) | Marine pill bug | consumer omnivore | [43] |
| *Tilapia zillii* | redbelly tilapia | consumer omnivore | [2] |
| [*Euchone limnicola*](http://www.marine.csiro.au/crimp/nimpis/spSummary.asp?txa=6465) | Fanworm | deposit feeder | [43] |
| *Neilo australis* | bivalve, New Zealand Nut Shell | deposit feeder | [9] |
| [*Polydora ciliata*](http://www.marine.csiro.au/crimp/nimpis/spSummary.asp?txa=6127) | Bristleworm | deposit feeder | [43] |
| *Polydora cornuta* | mud worm | deposit feeder | [9] |
| *Polydora websteri* | Boring Spionid | deposit feeder | [9] |
| *Tanais dulongi* | a tanaid | deposit feeder, detritivore | [9] |
| [*Boccardia proboscidea*](http://www.marine.csiro.au/crimp/nimpis/spSummary.asp?txa=6126) | Californian polydorid | deposit feeder, macroplanktivore | [43] |
| [*Laticorophium baconi*](http://www.marine.csiro.au/crimp/nimpis/spSummary.asp?txa=8578) | North American Pacific corophiid | deposit feeder, macroplanktivore | [43] |
| *Euplana gracilis* | a polyclad flatworm | detritivore | [81] |
| *Halicarcinus innominatus* | Pill-box crab | detritivore | [82] |
| *Nassarius kraussianus* | gastropod | detritivore | [83] |
| *Sphaeroma serratum* | an isopod | detritivore | [84] |
| *Tilapia mariae* | Spotted Tilapia | detritivore, herbivore, macroplanktivore | [44] |
| *Petrolisthes elongatus* | New Zealand half-crab | detritivore, macroplanktivore | [85] |
| *Paradexamine pacifica* | Dexaminid Amphipod | detritovore, herbivore | [86] |
| *Aplysiopsis formosa* | a nudibranch | herbivore | [9] |
| [*Paracerceis sculpta*](http://www.marine.csiro.au/crimp/nimpis/spSummary.asp?txa=6281) | Sponge isopod | herbivore | [43] |
| [*Paradella dianae*](http://www.marine.csiro.au/crimp/nimpis/spSummary.asp?txa=6282) | Sphaeromatid isopod | herbivore | [43] |
| *Phytia myosotis* | mouse ear ovatella | herbivore | [30] |
| *Poecilia latipinna* | sailfin molly | herbivore | [2] |
| *Zeacumantus subcarinatus* | a gastropod | herbivore | [87] |
| *Amphisbetia operculata* | a hydroid | macroplanktivore |  |
| *Anguinella palmata* | Bryozoan | macroplanktivore |  |
| [*Antennella secundaria*](http://www.marine.csiro.au/crimp/nimpis/spSummary.asp?txa=8132) | Knotted thread hydroid | macroplanktivore | [43] |
| *Aplysilla rosea* | a sponge | macroplanktivore |  |
| *Balanus improvisus* | Bay Barnacle | macroplanktivore |  |
| *Balanus patellaris* | a barnacle | macroplanktivore |  |
| *Balanus reticulatus* | a barnacle | macroplanktivore |  |
| [*Barentsia benedeni*](http://www.marine.csiro.au/crimp/nimpis/spSummary.asp?txa=10302) | Nodding head | macroplanktivore | [43] |
| *Bowerbankia gracilis* | Bryozoan | macroplanktivore |  |
| *Bowerbankia imbricata* | Bryozoan | macroplanktivore |  |
| [*Bugula flabellata*](http://www.marine.csiro.au/crimp/nimpis/spSummary.asp?txa=6931) | Bryozoan | macroplanktivore | [43] |
| [*Bugula neritina*](http://www.marine.csiro.au/crimp/nimpis/spSummary.asp?txa=6929) | Bryozoan | macroplanktivore | [43] |
| *Bugula simplex* | Bryozoan | macroplanktivore |  |
| *Bugula stolonifera* | Bryozoan | macroplanktivore |  |
| *Celleporella hyalina* | a bryozoan | macroplanktivore |  |
| [*Ciona intestinalis*](http://www.marine.csiro.au/crimp/nimpis/spSummary.asp?txa=6905) | Solitary ascidian | macroplanktivore | [43] |
| *Conopeum reticulum* | an encrusting bryozoan | macroplanktivore |  |
| [*Corbula gibba*](http://www.marine.csiro.au/crimp/nimpis/spSummary.asp?txa=6147) | European clam | macroplanktivore | [43] |
| [*Cordylophora caspia*](http://www.marine.csiro.au/crimp/nimpis/spSummary.asp?txa=10273) | Hydroid | macroplanktivore | [43] |
| [*Crassostrea gigas*](http://www.marine.csiro.au/crimp/nimpis/spSummary.asp?txa=6130) | Pacific oyster | macroplanktivore | [43] |
| [*Cryptosula pallasiana*](http://www.marine.csiro.au/crimp/nimpis/spSummary.asp?txa=6941) | Bryozoan | macroplanktivore | [43] |
| *Dysidea avara* | a sponge | macroplanktivore | [88] |
| *Dysidea fragilis* | a sponge | macroplanktivore |  |
| *Ectopleura dumortieri* | a cnidaria | macroplanktivore |  |
| *Electra pilosa* | a bryozoan | macroplanktivore |  |
| *Eudendrium carneum* | a hydroid | macroplanktivore |  |
| *Fenestrulina malusii* | a bryozoan | macroplanktivore |  |
| *Halecium vasiforme* | a hydrozoan | macroplanktivore |  |
| *Halisarca dujardini* | a sponge | macroplanktivore |  |
| *Hydroides diramphus* | a tube worm | macroplanktivore | [9] |
| *Hydroides ezoensis* | Tubeworm | macroplanktivore | [89] |
| *Hydroides sanctaecrucis* | Caribbean serpulid tubeworm | macroplanktivore | [9] |
| *Jassa marmorata* | Amphipod | macroplanktivore | [90] |
| [*Maoricolpus roseus*](http://www.marine.csiro.au/crimp/nimpis/spSummary.asp?txa=6150) | New Zealand screw shell | macroplanktivore | [43] |
| *Megabalanus occator* | Barnacle | macroplanktivore |  |
| [*Megabalanus rosa*](http://www.marine.csiro.au/crimp/nimpis/spSummary.asp?txa=7031) | Acorn barnacle | macroplanktivore |  |
| [*Megabalanus tintinnabulum*](http://www.marine.csiro.au/crimp/nimpis/spSummary.asp?txa=7023) | Acorn barnacle | macroplanktivore |  |
| *Megabalanus zebra* | Barnacle | macroplanktivore |  |
| *Molgula manhattensis* | Common Sea Grape | macroplanktivore |  |
| [*Monocorophium acherusicum*](http://www.marine.csiro.au/crimp/nimpis/spSummary.asp?txa=6458) | Mediterranean corophiid | macroplanktivore | [43] |
| [*Monocorophium insidiosum*](http://www.marine.csiro.au/crimp/nimpis/spSummary.asp?txa=6457) | English corophiid | macroplanktivore | [43] |
| *Monotheca obliqua* | a hydroid | macroplanktivore |  |
| [*Musculista senhousia*](http://www.marine.csiro.au/crimp/nimpis/spSummary.asp?txa=6146) | Bag mussel | macroplanktivore | [43] |
| [*Mytilopsis sallei*](http://www.marine.csiro.au/crimp/nimpis/spSummary.asp?txa=8064) | Black striped mussel | macroplanktivore | [43] |
| *Notomegabalanus algicola* | a barnacle | macroplanktivore |  |
| *Ostrea chilensis* | New Zealand flat oyster | macroplanktivore |  |
| *Ostrea edulis* | edible oyster | macroplanktivore |  |
| *Paphies ventricosa* | surf clam | macroplanktivore | [91] |
| *Perna canaliculus* | Green lipped mussel | macroplanktivore |  |
| [*Perna viridis*](http://www.marine.csiro.au/crimp/nimpis/spSummary.asp?txa=9492) | Asian green mussel | macroplanktivore | [43] |
| [*Raeta pulchella*](http://www.marine.csiro.au/crimp/nimpis/spSummary.asp?txa=6215) | Bivalve | macroplanktivore | [43] |
| [*Ruditapes largillierti*](http://www.marine.csiro.au/crimp/nimpis/spSummary.asp?txa=6149) | Venus clam | macroplanktivore | [43] |
| [*Sabella spallanzanii*](http://www.marine.csiro.au/crimp/nimpis/spSummary.asp?txa=6129) | European fan worm | macroplanktivore | [43] |
| *Schizoporella errata* | a bryozoan | macroplanktivore |  |
| [*Schizoporella unicornis*](http://www.marine.csiro.au/crimp/nimpis/spSummary.asp?txa=6943) | Single Horn Bryozoan | macroplanktivore |  |
| *Scruparia ambigua* | a creeping bryozoan | macroplanktivore |  |
| *Scrupocellaria bertholetti* | a bryozoan | macroplanktivore |  |
| *Scrupocellaria scruposa* | a bryozoan | macroplanktivore |  |
| [*Styela clava*](http://www.marine.csiro.au/crimp/nimpis/spSummary.asp?txa=6836) | Leathery sea squirt | macroplanktivore |  |
| *Tricellaria occidentalis* | a bryozoan | macroplanktivore |  |
| [*Tubularia crocea*](http://www.marine.csiro.au/crimp/nimpis/spSummary.asp?txa=6125) | Hydroid | macroplanktivore |  |
| [*Watersipora arcuata*](http://www.marine.csiro.au/crimp/nimpis/spSummary.asp?txa=6938) | Lace coral | macroplanktivore |  |
| *Watersipora subtorquata* | Lacy Bryozoan | macroplanktivore |  |
| *Zoobotryon verticillatum* | Bryozoan | macroplanktivore |  |
| *Bugula calathus* | Bryozoan | macroplanktivore (bugula neritina) |  |
| *Caprella californica* | Wood Boring Shipworm | macroplanktivore, detritivore | [92] |
| [*Teredo navalis*](http://www.marine.csiro.au/crimp/nimpis/spSummary.asp?txa=10036) | Naval shipworm | macroplanktivore, detritivore | [43] |
| *Pachypygus gibber* | parasitic copepod | parasite | [93] |
| [*Alexandrium minutum*](http://www.marine.csiro.au/crimp/nimpis/spSummary.asp?txa=6923) | Toxic dinoflagellate | phytoplankton | [43] |
| [*Gymnodinium catenatum*](http://www.marine.csiro.au/crimp/nimpis/spSummary.asp?txa=6927) | Toxic dinoflagellate | phytoplankton | [43] |
| *Neomysis japonica* | a mysid | planktivore |  |
| *Spartina anglica* | Common cord-grass | plant |  |
| *Oncorhynchus mykiss* | Rainbow trout | predator | [2] |
| *Oratosquilla oratoria* | a spearing mantis shrimp | predator | [94] |
| *Porichthys notatus* | plainfin midshipman | predator | [2] |
| *Salmo salar* | Atlantic salmon | predator | [2] |
| *Salmo trutta* | Brown trout | predator | [2] |
| *Salvelinus fontinalis* | brook trout | predator | [2] |
| *Sparidentex hasta* | Sobaity sea bream | predator | [2] |
|  |  |  |  |
