# Supplementary material for: Invasions and Extinctions Reshape Coastal Marine Food Webs
Source: PLoS One. 2007 Mar 14;2(3):e295. doi: 10.1371/journal.pone.0000295 (PMC1808429; doi:10.1371/journal.pone.0000295)
Supplement: Table S5 — List of marine species invasions in the Wadden Sea from Nehring (2006), their trophic group, and reference for trophic group from literature survey. Reference list follows in supplementary references S1. For a full list of all species in the Wadden Sea classified in trophic groups, please contact the authors. (0.11 MB DOC) [file pone.0000295.s005.doc]

# Supplementary Table S5

List of marine species invasions in the Wadden Sea from Nehring (2006), their trophic group, and reference for trophic group from literature survey. Reference list follows in supplementary references S1. For a full list of all species in the Wadden Sea classified in trophic groups, please contact the authors.

| Table S5: Invasions in the Wadden Sea | |  |  |
| --- | --- | --- | --- |
| **Species** | **Trophic Group** | **Reference** |  |
| *Bonnemaisonia hamifera* | Algae |  |  |
| *Colpomenia peregrina* | Algae |  |  |
| *Dasya baillouviana* | Algae |  |  |
| *Gracilaria vermiculophylla* | Algae |  |  |
| *Polysiphonia harveyi* | Algae |  |  |
| *Sargassum muticum* | Algae |  |  |
| *Eriocheir sinensis* | Consumer Omnivore | [32] |  |
| *Gammarus tigrinus* | Consumer Omnivore | [95] |  |
| *Rhithropanopeus harrisii* | Consumer Omnivore | [33] |  |
| *Ensis americanus* | Deposit Feeder | [96] |  |
| *Marenzelleria cf. viridis* | Deposit Feeder | [9] |  |
| *Marenzelleria cf. wireni* | Deposit Feeder | [9] |  |
| *Proasellus coxalis* | Detritivore | [97] |  |
| *Teredo navalis* | Detritivore | [96] |  |
| *Potamopyrgus antipodarum* | Herbivore | [96] |  |
| *Balanus improvisus* | Macroplanktivore |  |  |
| *Bimeria franciscana* | Macroplanktivore |  |  |
| *Congeria leucophaeta* | Macroplanktivore |  |  |
| *Cordylophora caspia* | Macroplanktivore |  |  |
| *Crassostrea gigas* | Macroplanktivore |  |  |
| *Crepidula fornicata* | Macroplanktivore | [9] |  |
| *Diadumene cincta* | Macroplanktivore |  |  |
| *Elminius modestus* | Macroplanktivore |  |  |
| *Ficopomatus enigmaticus* | Macroplanktivore | [9]; [98] |  |
| *Mya arenaria* | Macroplanktivore | [29] |  |
| *Nemopsis bachei* | Macroplanktivore |  |  |
| *Petricola pholadiformis* | Macroplanktivore | [96] |  |
| *Styela clava* | Macroplanktivore |  |  |
| *Victorella pavida* | Macroplanktivore |  |  |
| *Corophium curvispinum* | Macroplanktivore, Deposit Feeder | [9] |  |
| *Corophium sextonae* | Macroplanktivore, Deposit Feeder | [9] |  |
| *Caprella mutica* | Macroplanktivore, Detritivore | [29] |  |
| *Anguillicola crassus* | Parasite | [99] |  |
| *Chattonella antiqua* | Phytoplankton |  |  |
| *Chattonella marina* | Phytoplankton |  |  |
| *Coscinodiscus wailesii* | Phytoplankton |  |  |
| *Fibrocapsa japonica* | Phytoplankton |  |  |
| *Gymnodinium mikimotoi* | Phytoplankton |  |  |
| *Odontella (Biddulphia) sinensis* | Phytoplankton |  |  |
| *Thalassiosira punctigera* | Phytoplankton |  |  |
| *Acartia tonsa* | Planktivore |  |  |
| *Spartina anglica* | Plant |  |  |
