# Supplementary material for: Invasions and Extinctions Reshape Coastal Marine Food Webs
Source: PLoS One. 2007 Mar 14;2(3):e295. doi: 10.1371/journal.pone.0000295 (PMC1808429; doi:10.1371/journal.pone.0000295)
Supplement: References S1 — Reference list for trophic classification from literature survey of all species in supplementarytables S1, S2, S3, S4 and S5. (0.08 MB DOC) [file pone.0000295.s006.doc]

#### Supplementary References S1

#### Reference list for trophic classification from literature survey of all species in supplementary tables S1-S5.
